# Do not attempt resuscitation decisions in a cancer centre: addressing difficult ethical and communication issues

**DOI:** 10.1038/sj.bjc.6600205

**Published:** 2002-04-08

**Authors:** C Reid, D Jeffrey

**Affiliations:** Three Counties Cancer Network, Cheltenham General Hospital, Cheltenham G53 7AN, UK

**Keywords:** resuscitation, do not attempt resuscitation orders, communication skills

## Abstract

Talking to patients about ‘Do Not Attempt Resuscitation’ decisions is difficult for many doctors. Communication about ‘Do Not Attempt Resuscitation’ decisions should occur as part of a wider discussion of treatment goals at an earlier stage in the patient's illness. A doctor should not initiate any treatment, including cardio-pulmonary resuscitation if he/she does not believe it will benefit the patient. An ethical framework is offered which may be of practical help in clarifying decision-making.

*British Journal of Cancer* (2002) **86**, 1057–1060. DOI: 10.1038/sj/bjc/6600205 www.bjcancer.com

© 2002 Cancer Research UK

## 

Doctors and nurses commonly encounter ethical dilemmas surrounding issues of cardiopulmonary resuscitation (CPR) and do not attempt resuscitation (DNAR) decisions. Recent guidelines from the British Medical Association (BMA), Resuscitation Council (UK) and the Royal College of Nursing on DNAR decisions are welcome (British Medical Association *et al*, 2001). However, they do not resolve some of the most difficult dilemmas, which relate to communication issues with patients and their relatives. In this paper we suggest that communication about DNAR orders may be improved if they are not seen as separate consultations but discussed with the patient within the context of treatment plans. We also consider situations when it is not appropriate to discuss such decisions with patients and provide an ethical framework to aid the medical team. Our discussion relates particularly to our clinical practice with patients in a Cancer Centre, whose prognosis and aims of treatment vary. Some patients are receiving treatment with a good prospect of cure or prolonged remission. Others may be incurable and are receiving palliative treatments with an uncertain prognosis, or may be in a terminal phase where life expectancy is no more than days.

## EXPECTATIONS OF CPR

In the last 30 years CPR has evolved from an intervention developed to treat the reversible precipitants of sudden death, indicated only in cases of acute insult to an otherwise healthy person, to a default measure employed in virtually all cases of death in hospital (Willard, 2000). Confusion exists, both in the minds of patients and doctors, between ‘normal death’ and cardio respiratory arrest. Moreover, an increasing patient expectation of survival, combined with fears about hospitals and a lack of trust in doctors, has perhaps contributed to the excessive use of CPR. Society and the medical profession seem to have forgotten that death is an integral part of life (Smith, 2000).

A review of the literature reveals that most patients want their views taken into account in determining their treatment options but feel that doctors should be the main decision makers (Kerridge *et al*, 1998; Downes and Liddle, 2001). With regard to CPR, patients and relatives lack knowledge of the process involved (Agard *et al*, 2000; Brett *et al*, 2001). To make autonomous judgments patients need better education on CPR outcomes, which may be achieved by supplementing discussion with written information (Kerridge *et al*, 1999). Respect for autonomy restricts CPR use when it is refused by the patient but cannot create a ‘right to CPR’ (Luce, 1995; Hilberman *et al*, 1997; Willard, 2000).

The poor survival rates documented following in-hospital cardiac arrests suggests that unsuccessful CPR is common (Marik and Craft, 1997; De Vos *et al*, 1999; Rien de Vos *et al*, 1999). Such practice may be emotionally fraught and demoralising for hospital teams. In the USA, hospital practitioners fully respect patient autonomy by seeking the patient's or surrogate's consent in every instance. However such an approach grants doctors no authority to refuse to render treatments that are in their judgments contraindicated (Mello and Jenkinson, 1998). This practice can be contrasted with that found in the UK, where practices vary depending on the patient's situation. For example, in general, CPR is not carried out in hospices or at home, whereas DNAR decisions are routinely considered in general and oncology wards although they may not be discussed with the patient (Willard, 2000). Thus doctors in the UK are enabled to reduce the inappropriate use of resuscitation, but at the expense of patient autonomy (Mello and Jenkinson, 1998).

## GUIDELINES

Locally agreed policies on CPR and DNAR decisions are necessary to enhance clinical care, to respect the autonomy and dignity of patients and to protect individual doctors and nurses from criticism. The recent BMA, Resuscitation Council and RCN guidelines are helpful in that they ‘outline legal and ethical standards for planning patient care and decision-making in relation to cardiopulmonary resuscitation’ (British Medical Association *et al*, 2001). They aim to demystify the process by which decisions are made by emphasising the need to discuss the reasons for the DNAR order with the patient.

## COMMUNICATION PROBLEMS

Talking about DNAR decisions with patients and relatives is not easy. Doctors may lack the necessary communication skills and may feel a sense of failure or fear litigation. They may worry that the patient will be distressed by the discussion. Another difficulty for doctors is that the discussion usually takes place when CPR is being withdrawn as a treatment option. There is also the question as to which doctor in the team should discuss these issues with the patient and their relatives. This question relates not just to the seniority or experience of the physician but also to their communication skills and availability. Communication about these complex issues should be viewed as a process, which may not be completed in a single consultation. Patient recall of DNAR discussions may be poor and doctors need to be aware of the need to give patients an opportunity to review their decision if they so wish (Stewart and Spice, 2001).

## IMPROVING THE COMMUNICATION OF DNAR DECISIONS

Recent guidelines aim to demystify the process of DNAR orders for patients and to promote trust (British Medical Association *et al*, 2001). The decision-making guidelines also recommend ‘advanced planning for anticipated medical events’ (British Medical Association *et al*, 2001). We propose that if DNAR discussions took place within the context of discussions around aims of treatment at earlier hospital admissions, for example for chemotherapy or treatment of neutropenic sepsis, some patients would then have opportunities to discuss CPR at a time when it *was* considered an appropriate option. As disease progresses, the opportunity to discuss changing aims of treatment, quality of life issues and expectations of the future should be taken in order to facilitate understanding and acceptance of a DNAR order when this becomes appropriate.

The critical issues in improving communication around DNAR orders lie in the timing of the discussion and in ensuring that the conversation relating to CPR forms part of a wider range of negotiations, which explain the aims of care. In this way, the patient gains insight and understanding that their disease is progressing. They come to appreciate that the goals of care are now comfort, improving quality of life and maintaining dignity. Consequently they understand that interventions such as surgery, chemotherapy or CPR are no longer appropriate.

It is clear that many patients and relatives do not understand the nature of CPR and DNAR orders (Agard *et al*, 2000; Brett *et al*, 2001). It is important to explain that this technique evolved in response to sudden cardiac arrest rather than as an intervention to be used in advanced cancer. It is also important to stress the supportive care the medical teams do feel is appropriate e.g. intravenous (i.v.) antibiotics for sepsis, i.v. hydration and optimal symptom control. This should be documented in the patient's notes alongside the DNAR order. Otherwise, patients and their relatives may fear that a DNAR order will adversely affect the quality of their care (Ebrahim, 2000). It is the doctor's responsibility to ensure this is never the case.

Although relatives have no legal right to make decisions for another adult, staff should be available to discuss and explain the reasons why a DNAR decision would be appropriate to protect an individual from unhelpful and potentially undignified treatment and also to explain that all other treatments to ensure the comfort and dignity of the patient would still be carried out (Luce, 1995).

As DNAR discussions became more commonplace, we would hope that less experienced doctors would be trained in the necessary communication techniques and the palliative care team would have an educational role here. Our experience is that the hospital palliative care teams could also be involved at the oncology – palliative care interface when such discussions take place and their role would be to support the patient, family and health professionals involved. Occasionally, it may even be appropriate for the palliative care team to initiate the discussion with the patient and to convey the outcome to the oncology team., However, we have identified key practical difficulties, which are not addressed by published guidelines:

- The patient demands CPR when the medical team feel it would not be beneficial
- The role of the family
- Notions of futility

## THE PATIENT DEMANDS CPR

The guidelines do not clarify what the doctor should do if the patient refuses a DNAR order (British Medical Association *et al*, 2001). Can a doctor be compelled to offer a treatment, which he or she feels, will not benefit a patient? It is generally accepted that doctors have no legal obligation to offer any treatment, which in their professional judgment will not be beneficial (Luce, 1995). However, the guidelines are ambiguous, stating that ‘doctors cannot be required to give treatment contrary to their clinical judgment but should, whenever possible, respect patients' wishes to receive treatment which carries only a very small chance of success or benefit’ (British Medical Association *et al*, 2001). Perhaps it is the fear of having a patient refuse a DNAR order that causes doctors to delay discussions until the patient is dying, when he/she may be unable to participate in decision-making. Doctors need to be able to discuss with patients that a treatment is not appropriate when it is unlikely to confer benefit or when it is likely to cause more harm than good. Refusing a treatment may be difficult for doctors particularly when it may appear that ‘life is at stake’. However, in patients with advanced cancer, successful CPR rarely prolongs life for more than a short time, and does not improve its quality. Attempting CPR in such situations is medically inappropriate and promotes the myth that doctors can postpone death indefinitely. In our experience, given time and the opportunity for discussions, it is extremely rare for a patient to continue to insist on attempts at resuscitation.

## THE ROLE OF THE FAMILY

When the patient is no longer competent to make decisions, the family is involved in discussions and they may have strong views as to whether resuscitation should, or should not, be attempted. The guidelines make it clear that when doctors are talking to the family, they are seeking clarification of what the patient would have wished for, rather than what the relatives themselves would wish. Many relatives do not share this perception and feel that they have a right to decide whether to consent to or refuse CPR. This can lead to conflict with the medical staff or leave relatives with feelings of guilt in bereavement.

## NOTIONS OF FUTILITY

Notions of the futility of CPR risk obscuring the decision-making process. Clearly, if the patient were dying from a terminal illness, CPR would be futile and inappropriate. (Ebell, 1992; Marik and Craft, 1997; Ebell *et al*, 1998; De Vos *et al*, 1999; Gwinnutt *et al*, 2000). Notions of futility are centred on whether CPR is likely to be successful. It is more helpful for the doctor to make a judgment about whether successful CPR is likely to benefit the patient. Such judgment would need to take into account whether the patient is likely to achieve a level of recovery, which justifies the potential burdens of the treatment.

## RECOMMENDATIONS FOR IMPROVING INTERPROFESSIONAL COMMUNICATION

DNAR decisions should be recorded in the medical notes in a manner that reflects the professional's respect for the patient's dignity. It should be noted that there has been a clinical assessment by a senior clinician (consultant or registrar) with responsibility for the patient's care and that attempts at resuscitation would not benefit the patient. The note should include the nature of the discussion with the patient and the family, the diagnosis, the time, the date of review, the date the order was made and the signature of the responsible physician. If such discussion was either not appropriate or possible the reasons for this must be stated (Box 1

**Box 1:**
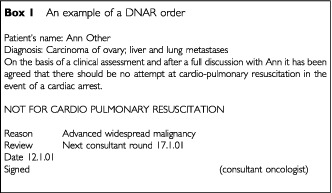
Box 1. An example of a DNAR order

).

Any form of jargon or medical shorthand is totally unacceptable. The decision must be shared between the medical and nursing teams. Procedures within teams for making decisions should be clear and the views of the whole team need to be explored (British Medical Association *et al*, 2001). It is helpful if both medical and nursing staff can be present when discussing these issues with the patient.

Just as a signed consent form is an acknowledgement that a process of informed consent has taken place, so too the documentation in the notes of a DNAR order should be a record that the process of decision-making has been carried out in the appropriate way.

We think that there are many benefits of improving communication in this way. Perhaps the most important is that full informed consent is achieved for the DNAR order; although it is unusual to obtain informed consent for a treatment being withheld. The result is a more open and honest relationship with the patient and their family. All parties, including on-call medical teams, are made aware of aims of treatment and measures that are still felt to be appropriate.

## A MODEL FOR ETHICAL DECISION-MAKING AROUND DNAR ORDERS

A new way of approaching the issue of DNAR orders brings CPR into the everyday discussion with the patient while they are competent but as the disease progresses, and the likely benefit of CPR becomes negligible, the ethical case for the doctor taking the responsibility for the decision becomes stronger.

**Figure 1:**
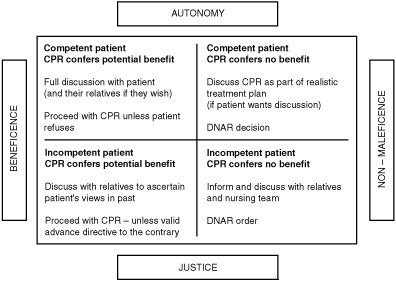
An ethical approach to DNAR decision-making.

In thinking about ethical dilemmas the principles of the duties of respect for autonomy, beneficence, non-maleficence and justice are useful (Beauchamp and Childress, 1994). In a clinical situation these duties often conflict and Figure 1

illustrates a way in which the ethical duties might be given differing weights in differing clinical situations and may clarify decision-making. Doctors must ensure that each patient is considered as an individual.

## DISCUSSION

Is it ever ethical not to discuss a DNAR decision with a competent patient? Whilst there is a strong ethical presumption in favour of discussing treatment decisions with patients, DNAR is a ‘withholding of treatment’ so requires special attention. If, in the judgment of the senior doctor, the patient may benefit from CPR then it is mandatory to discuss this with the patient. If however, in the doctor's professional judgment, CPR would not benefit the patient, as a consequence of the severity of their underlying clinical condition, and the doctor feels that the harm of discussion outweigh the benefits, then there is no legal or moral imperative to discuss CPR with the patient (Luce, 1995; Dunphy, 2000).

Although generally we would advocate earlier discussion about CPR with competent patients, in dying patients, a DNAR decision may be made by the responsible consultant without the competent patient's involvement (Downes and Liddle, 2001). However, doctors may issue DNAR orders without discussion with the patient for other reasons; fear of causing distress, fear of refusal of a DNAR order; conflict with the family and confusion surrounding futility. In such cases where it would be appropriate to have discussions with the patient, doctors may be more likely to discuss CPR and DNAR issues if they have become accustomed to the discussion and if they have received appropriate communication skills teaching. Even so, some decisions will be difficult and may result in conflict between doctors and patients, doctors and the family or between professionals. In such situations our experience is that the support of the specialist palliative care team may be of value.
